# A New Series of Pyrrole-Based Chalcones: Synthesis and Evaluation of Antimicrobial Activity, Cytotoxicity, and Genotoxicity

**DOI:** 10.3390/molecules22122112

**Published:** 2017-11-30

**Authors:** Ahmet Özdemir, Mehlika Dilek Altıntop, Belgin Sever, Hülya Karaca Gençer, Handan Açelya Kapkaç, Özlem Atlı, Merve Baysal

**Affiliations:** 1 Department of Pharmaceutical Chemistry, Faculty of Pharmacy, Anadolu University, Eskişehir 26470, Turkey; mdaltintop@anadolu.edu.tr (M.D.A.); belginsever@anadolu.edu.tr (B.S.); 2 Department of Pharmaceutical Microbiology, Faculty of Pharmacy, Anadolu University, Eskişehir 26470, Turkey; hulyakaraca@anadolu.edu.tr; 3 Department of Biology, Faculty of Science, Anadolu University, Eskişehir 26470, Turkey; haakdamar@anadolu.edu.tr; 4 Department of Pharmaceutical Toxicology, Faculty of Pharmacy, Anadolu University, Eskişehir 26470, Turkey; oatli@anadolu.edu.tr (Ö.A.); mbaysal@anadolu.edu.tr (M.B.)

**Keywords:** antimicrobial activity, chalcone, cytotoxicity, furan, genotoxicity, pyrrole

## Abstract

In an effort to develop new potent antimicrobial and anticancer agents, new pyrrole-based chalcones were designed and synthesized via the base-catalyzed Claisen-Schmidt condensation of 2-acetyl-1-methylpyrrole with 5-(aryl)furfural derivatives. The compounds were evaluated for their in vitro antimicrobial effects on pathogenic bacteria and *Candida* species using microdilution and ATP luminescence microbial cell viability assays. MTT assay was performed to determine the cytotoxic effects of the compounds on A549 human lung adenocarcinoma, HepG2 human hepatocellular carcinoma, C6 rat glioma, and NIH/3T3 mouse embryonic fibroblast cell lines. 1-(1-Methyl-1*H*-pyrrol-2-yl)-3-(5-(4-chlorophenyl)furan-2-yl)prop-2-en-1-one (**7**) and 1-(1-methyl-1*H*-pyrrol-2-yl)-3-(5-(2,5-dichlorophenyl)furan-2-yl)prop-2-en-1-one (**9**) were found to be the most potent antifungal agents against *Candida krusei* and therefore these compounds were chosen for flow cytometry analysis and Ames MPF assay. ATP bioluminescence assay indicated that the antifungal activity of compounds **7** and **9** against *C. krusei* was significantly higher than that of other compounds and the reference drug (ketoconazole), whereas flow cytometry analysis revealed that the percentage of dead cells treated with compound **7** was more than that treated with compound **9** and ketoconazole. According to Ames MPF assay, compounds **7** and **9** were found to be non-genotoxic against TA98 and TA100 with/without metabolic activation. MTT assay indicated that 1-(1-methyl-1*H*-pyrrol-2-yl)-3-(5-(2-nitrophenyl)furan-2-yl)prop-2-en-1-one (**3**) showed more selective anticancer activity than cisplatin against the HepG2 cell line. On the other hand, 1-(1-methyl-1*H*-pyrrol-2-yl)-3-(5-(4-nitrophenyl)furan-2-yl)prop-2-en-1-one (**1**) was found to be more effective and selective on the A549 cell line than cisplatin.

## 1. Introduction

Infections caused by pathogenic bacteria represent a major public health burden, not just in terms of morbidity and mortality, but also in terms of increased healthcare costs [1]. The treatment of bacterial infections is increasingly complicated by the ability of bacteria to rapidly evolve resistance to antimicrobial agents [1,2]. Opportunistic fungal infections, particularly those caused by *Candida* spp., have emerged as major causes of morbidity and mortality in immunocompromised patients. Fungi are eukaryotic pathogens, therefore the close evolutionary relationship between fungi and their human hosts has limited the number of drug targets that can be exploited to selectively kill the pathogen with minimal host toxicity [3,4,5,6]. On the other hand, cancer has become the leading cause of death in developed countries [7]. Over the years, the design of chemotherapy has become considerably sophisticated, yet there is no cancer treatment that is 100% effective against disseminated cancer [8,9]. As a result, the discovery and development of potent and selective anti-infective and anticancer agents has been the subject of considerable research in medicinal chemistry.

Chalcones play a pivotal role in the flavonoid biosynthetic pathway and are ubiquitously present in natural products, including many dietary products like fruits, vegetables, spices, and tea. Therapeutic applications of chalcones trace back thousands of years through the use of plants and herbs for the treatment of various diseases, such as cancer, inflammation, and diabetes. In particular, naturally occurring chalcones and their synthetic analogues have attracted a great deal of interest as potent candidates for the treatment of cancer due to their high therapeutic index, negligible side effects, and ease of synthesis. Chalcones have been reported to exert cytotoxic activity against many cancer cells through multiple mechanisms including cell cycle disruption, angiogenesis inhibition, apoptosis induction, tubulin polymerization inhibition, blockade of the NF-κB signalling pathway, and inhibition of cell cycle regulatory kinases [10,11,12,13,14,15]. On the other hand, several studies have pointed out the antimicrobial potential of chalcones against a wide range of fungi and bacteria, including resistant ones, clearly indicating that they are attractive target compounds for the discovery and development of new anti-infective agents [16,17,18,19,20,21,22].

In the drug discovery process, three-pronged strategies, namely structural manipulation of both aryl rings, replacement of aryl rings with heteroaryl scaffolds, and molecular hybridization through conjugation with biologically active scaffolds, are employed to enhance the biological activity of chalcones. The successful application of these three-pronged strategies for the discovery of new chalcone-based chemotherapeutic agents has resulted in chemically diverse chalcones with potential therapeutic applications in the treatment of cancer and infectious diseases [15,16].

**Figure 1:**
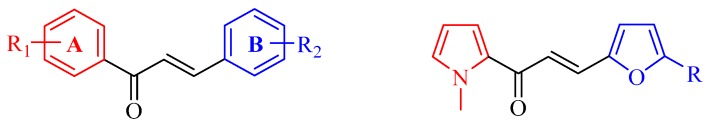
The design of new chalcone derivatives through the replacement of aryl rings (**A** and **B**).

In the current work, new chalcone derivatives were designed as potential antimicrobial and anticancer agents through the replacement of aryl rings (**A** and **B**) with biologically active heterocyclic scaffolds, namely pyrrole and furan rings, to enhance biological activity (Figure 1). For this purpose, new chalcone derivatives were synthesized and screened for their in vitro antimicrobial effects on pathogenic bacteria and *Candida* species, and their genotoxic and cytotoxic effects on A549 human lung adenocarcinoma, HepG2 human hepatocellular carcinoma, C6 rat glioma, and NIH/3T3 mouse embryonic fibroblast cell lines.

## 2. Results and Discussion

### 2.1. Chemistry

**Figure 2:**
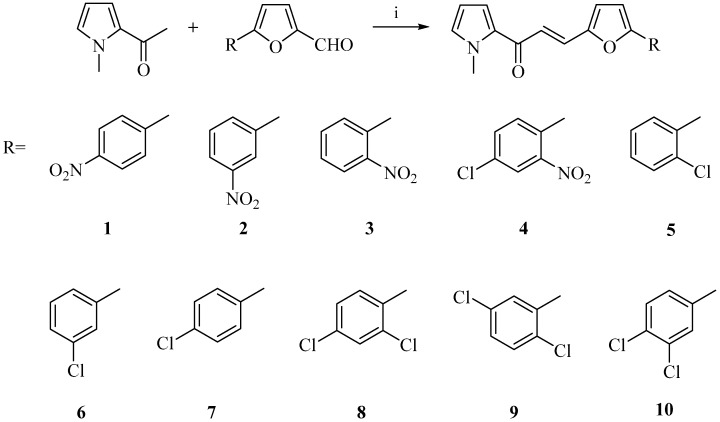
The synthetic route for the preparation of the chalcone derivatives (**1**–**10**). Reagents and conditions: (i) NaOH, methanol, r.t., 48 h.

The synthesis of the target compounds (**1**–**10**) was carried out as outlined in Figure 2. The base-catalyzed Claisen-Schmidt condensation of 2-acetyl-1-methylpyrrole with 5-(aryl)furfural derivatives afforded the chalcone derivatives (**1**–**10**). The reactions were carried out with 57–83% yield. The structures of the obtained compounds were elucidated using spectral data. 

In the IR spectra, the characteristic C=O stretching bands due to the carbonyl group were observed at about 1635.64–1649.14 cm^−1^. In the ^1^H-NMR spectra of the compounds, a sharp singlet was observed at 3.92–3.98 ppm, integrating for three protons that correspond to the 1*H*-pyrrole-C*H_3_* protons. The doublets appeared in the region 7.07–7.16 ppm (*J* = 3.20–4.80 Hz), corresponding to the furan C_4_-*H*. The doublets or broad singlets appeared in the region 7.17–7.24 ppm (*J* = 3.20–4.80 Hz), corresponding to the furan C_3_-*H*. The olefinic protons (-CO-C*H*=C*H*-) resonated as doublets (*J* = 15.20–15.60 Hz) and multiplets at 7.32–7.67 ppm. All the other aromatic protons were observed as expected. In the ^13^C-NMR spectra of the compounds, the signal of the characteristic carbonyl carbon appeared in the region 177.51–178.04 ppm. 1*H*-pyrrole-*C*H_3_ signals were observed at 37.14–37.18 ppm.

In the mass spectra, the electrospray ionization (ESI) technique with positive ion mode was applied and M + 1 peaks were detected as base peaks.

### 2.2. Microbiology

Compounds **1**–**10** were screened for their in vitro antimicrobial effects on pathogenic bacteria and *Candida* species using microdilution susceptibility assay. Generally, the compounds were more effective on fungi than the bacteria used in this study (Table 1). According to the minimum inhibitory concentration (MIC) results, compounds **2** and **10** were the most effective antibacterial agents in this series on *Enterococcus faecalis* (ATCC 51299) with a MIC value of 100 μg/mL when compared with chloramphenicol (MIC = 100 μg/mL). On the other hand, compounds **3** and **7** were the most potent antifungal agents in this series against *Candida albicans* with a MIC value of 50 μg/mL when compared with ketoconazole (MIC = 50 μg/mL). Compounds **2**, **3**, **4**, **7**, **8**, and **9** were found as the most active compounds against *Candida glabrata* with a MIC value of 100 μg/mL. Compounds **3**, **4**, and **9** also showed notable anticandidal activity against *Candida parapsilosis* with a MIC value of 50 μg/mL. Compound **8** and ketoconazole displayed antifungal activity against *Candida krusei* with a MIC value of 50 μg/mL. In addition, compounds **7** and **9** were more effective than ketoconazole on *C. krusei*. 4-Chloro substituted compound **7** and 2,5-dichloro substituted compound **9** showed anticandidal activity against *C. krusei* with a MIC value of 25 μg/mL. This outcome pointed out the importance of the position of chloro substituent for anticandidal activity. The antimicrobial efficiency of these compounds was further investigated using ATP bioluminescence assay as a more sensitive method [23].

According to microdilution susceptibility and ATP bioluminescence assays, none of the compounds were effective on *Staphylococcus aureus* and *Escherichia coli*. Both methods demonstrated the same results for *E. faecalis* (ATCC 51299) and compounds **2** and **10** showed antimicrobial activity close to chloramphenicol. Although the MIC results did not indicate any significant activity for *E. faecalis* (ATCC 29212), surprisingly, ATP bioluminescence assay showed that *E. faecalis* (ATCC 29212) was sensitive to compounds **1**, **2**, **3**, and **7** but not as much as chloramphenicol. Vancomycin resistance and high-level aminoglycoside resistance of *E. faecalis* (ATCC 51299), and susceptibility of *E. faecalis* (ATCC 29212) to vancomycin, gentamicin, and streptomycin are well known [24]. The discovery of new antimicrobial agents against *Enterococci* is highly important due to increasing mortality risk. For both *Enterococci* strains, in particular the chemical structure of compound **2** can be re-evaulated to design new, more effective antimicrobial agents. On the other hand, the compounds did not show any meaningful growth inhibitory effects on *K. pneumoniae* according to microdilution assay, whereas all compounds except compounds **5**, **6**, and **9** have antimicrobial activities close to chloramphenicol when considering the ATP luminescence assays.

**Figure 3:**
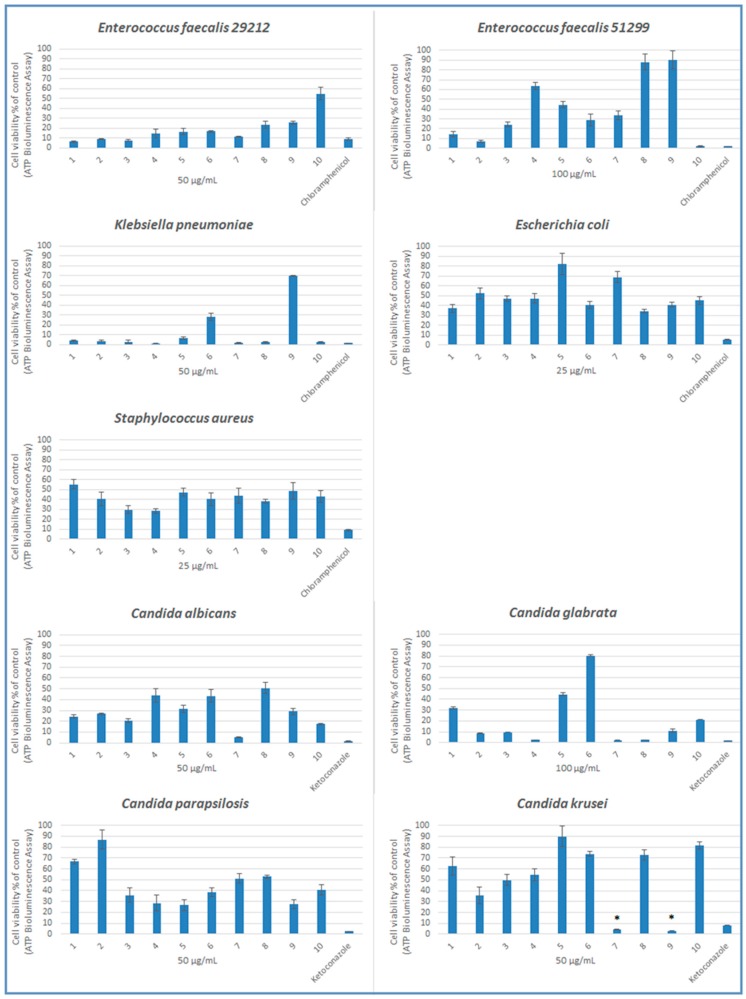
Effects of compounds **1**–**10** on cell viability. Bacteria and yeasts were treated for 24 h with compounds. Data are expressed as a percentage of untreated controls (which is set at 100%). Reference drugs are chloramphenicol for bacteria and ketoconazole for yeasts. * *p* ≤ 0.05. The results represent the mean ± SD of three independent experiments.

According to ATP bioluminescence assay results, compounds **7** and **9** have more statistically significant antifungal activity against *C. krusei* than ketoconazole (Figure 3). Compounds **4**, **7**, and **8** showed antifungal activity close to ketoconazole on *C. glabrata* regarding both methods. Unlike the microdilution assay, ATP bioluminescence assay indicated that none of the compounds showed antifungal activity against *C. parapsilosis*. In microdilution assay, compounds **3** and **7** showed more antifungal activity against *C. albicans* than other compounds, whereas these compounds showed no statistically significant effects in ATP bioluminescence assay. The reason for the false negative ATP bioluminescence assay results could be explained by the fact that several classes of inhibitory compounds interfere with luciferase enzyme activity. Inhibition of luciferase enzyme gives low relative light unit (RLU) values [25].

**Figure 4:**
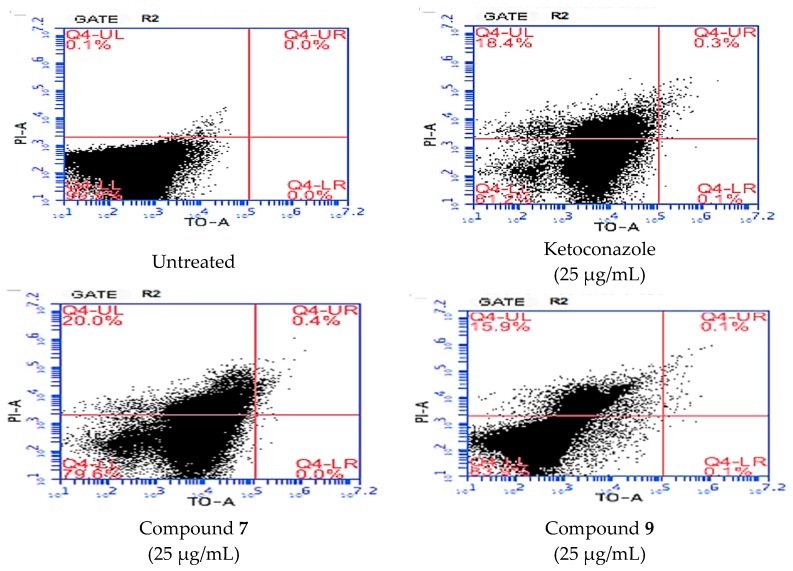
Flow cytometry analysis of *C. krusei* treated with compounds **7** and **9**. TO: Thiazole orange, PI: propidium iodide. Untreated sample was used as a control and gated according to the untreated yeast.

In order to evaluate the flow cytometry results, we focused on *C. krusei* since the MIC results of compounds **7** and **9** on *C. krusei* were statistically significant in this series. For this reason, *C. krusei* cells were treated with 25 µg/mL of compounds **7** and **9** which was found as the lowest effective concentration according to the microdilution assay. The percentage of inhibited cells was determined using flow cytometry. The results indicated that the activity of the compounds is very close to ketoconazole and there were only ~2% differences (Table 2). Contrary to the aforementioned assays, flow cytometry analysis of compound **9** showed less inhibitory activity than ketoconazole (Figure 4).

### 2.3. Cytotoxicity

Compounds **1**–**10** were evaluated for their anticancer activities against A549 human lung adenocarcinoma, HepG2 human hepatocellular carcinoma, and C6 rat glioma cell lines (Table 3).

Compounds **3** (IC_50_ = 27 μg/mL), **5** (IC_50_ = 31 μg/mL), and **7** (IC_50_ = 23 μg/mL) showed notable anticancer activity against the HepG2 cell line when compared with cisplatin (IC_50_ = 38 μg/mL). On the other hand, all compounds were found to be less potent than cisplatin against the C6 cell line. However, compounds **1** (IC_50_ = 84 μg/mL) and **8** (IC_50_ = 90 μg/mL) were the most effective agents in this series against the C6 cell line. When the compounds were compared in terms of their anticancer activity against the A549 cell line, compound **1** was identified as the most promising anticancer agent in this series with an IC_50_ value of 0.3 μg/mL, indicating that compound **1** was more effective than cisplatin (IC_50_ = 19 μg/mL). It is noteworthy to indicate that the *p*-nitro substituent significantly enhanced anticancer activity against the A549 cell line.

The cytotoxicity of anticancer agents against healthy cells limits their efficacy. An ideal anticancer drug should exert selectivity to cancer cells without causing any harm to healthy cells [26,27]. For this purpose, the selectivity of the compounds was tested against NIH/3T3 cell lines (Table 3). Compounds **7** and **9**, potent anticandidal agents against *C. krusei* in this series, showed IC_50_ values against NIH/3T3 cell line higher than their MIC values. These results pointed out the importance of these compounds as promising anticandidal agents against *C. krusei*.

Selectivity Index (SI), which is an important marker in comparing the safety and therapeutic value of the compounds, plays a key role in the drug development process [26,27]. Compounds with SI values higher than cisplatin can be considered as anticancer drug candidates. Compound **3**, having a SI value higher than cisplatin, was found to be the most potent anticancer agent against the HepG2 cell line in this series, therefore compound **3** can be considered as a promising candidate for further studies (Table 4). On the other hand, the SI value of compound **1** was significantly higher than that of cisplatin against the A549 cell line, indicating its significance as a promising anticancer candidate for the treatment of lung cancer, and should undergo further studies.

### 2.4. Genotoxicity

**Figure 5:**
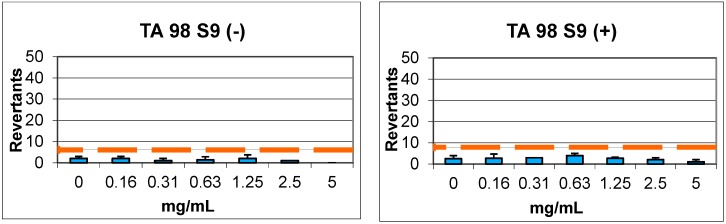

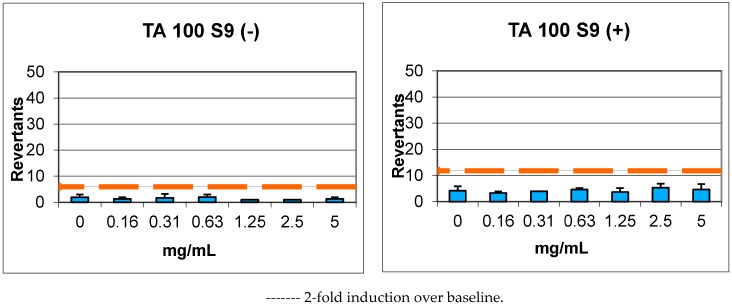
Ames MPF test results of compound **7** against TA98 and TA100 with/without S9.

In Ames microplate format (MPF) assay, compound **7** was calculated to have a baseline of 3.95 and 3.04, and did not show 1.5–2.5-fold increases over the baseline and any statistical significance in the tested doses against TA98 with/without S9 enzyme, respectively. Furthermore, the same compound showed a baseline of 3.00 and 5.91, and did not show 1.5–2.5-fold increases over the baseline and any statistical significance in the tested doses against TA100 with/without S9 enzyme, respectively (Figure 5, Table 5).

**Figure 6:**
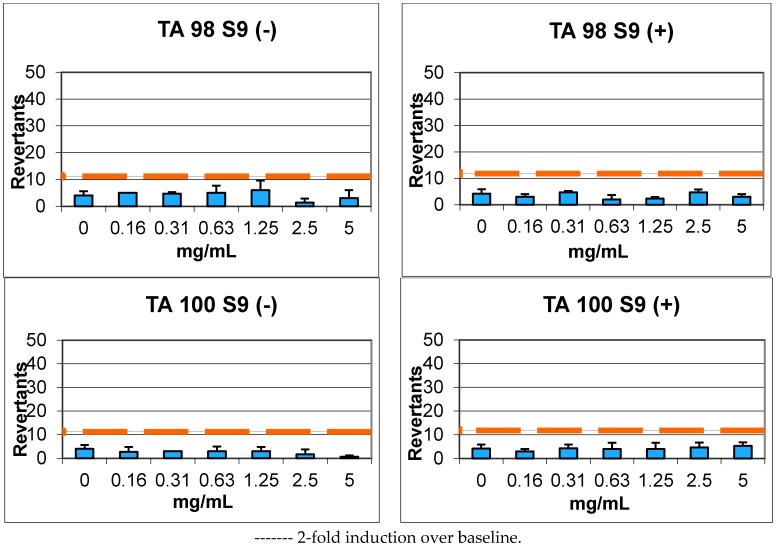
Ames MPF test results of compound **9** against TA98 and TA100 with/without S9.

Compound **9** showed a baseline of 5.92 against TA98 with S9 enzyme activation and 5.60 without S9 enzyme activation. In this case, 1.5–2.5-fold increases over the baseline and significant increases in a dose-dependent manner were not exhibited. According to the results, compound **9** was accepted as non-genotoxic in the tested doses. Compound **9** did not have 1.5–2.5-fold increases, as indicated in the criteria; the significant increases obtained with 2.5 mg/mL and 5 mg/mL concentrations were below the mentioned fold increases and not dose-dependent (Figure 6, Table 5).

In conclusion, compounds **7** and **9** were found to be non-genotoxic against TA98 and TA100 with/without metabolic activation according to the Ames results, indicating that these compounds were potential candidates for further studies.

## 3. Materials and Methods

### 3.1. Chemistry

All reagents were purchased from commercial suppliers and were used without further purification. The melting points (m.p.) of the compounds were determined on an Electrothermal 9100 melting point apparatus (Weiss-Gallenkamp, Loughborough, UK) and are uncorrected. IR spectra were recorded on an IRPrestige-21 Fourier Transform Infrared spectrophotometer (Shimadzu, Tokyo, Japan). ^1^H-NMR and ^13^C-NMR spectra were recorded on a Bruker spectrometer (Bruker, Billerica, MA, USA). Chemical shifts were reported in parts per million (ppm) and the coupling constants (*J*) were expressed in Hertz (Hz). Mass spectra were recorded on a Shimadzu LCMS-IT-TOF system (Shimadzu, Kyoto, Japan). Thin Layer Chromatography (TLC) was performed on TLC Silica gel 60 F254 aluminium sheets (Merck, Darmstadt, Germany) to check the purity of the compounds. 

General Procedure for the Synthesis of 1-(1-methyl-1*H*-pyrrol-2-yl)-3-(5-(aryl)furan-2-yl)prop-2-en-1-one Derivatives (**1**–**10**):

A mixture of 2-acetyl-1-methylpyrrole (2.5 mmol), 5-(aryl)furfural (2.5 mmol), and 50% (*w*/*v*) sodium hydroxide (2 mL) in methanol (20 mL) was stirred at room temperature for about 2 days. The progress of the reaction was checked by TLC. Upon completion, the reaction mixture was poured into crushed ice. The precipitated solid was filtered, washed with water, and dried. The product was crystallized from ethanol.

*1-(1-Methyl-1H-pyrrol-2-yl)-3-(5-(4-nitrophenyl)furan-2-yl)prop-2-en-1-one* (**1**). Yield: 81%; m.p. 179–181 °C. IR ν_max_ (cm^−1^): 3115.04 (aromatic C–H stretching), 2939.52 (aliphatic C–H stretching), 1637.56 (C=O stretching), 1583.56, 1558.48, 1508.33, 1475.54, 1456.26 (C=C stretching), 1404.18, 1381.03, 1327.03, 1300.02, 1246.02, 1211.30, 1107.21, 1062.78, 1045.42, 1031.92 (C–N and C–O stretching), 989.48, 964.41, 921.97, 850.61, 792.74, 746.45, 725.23, 686.66 (aromatic C–H out-of-plane bending). ^1^H-NMR (400 MHz, *δ* ppm, DMSO-*d*_6_): 3.93 (3H, s, CH_3_), 6.21 (1H, dd, *J* = 2.40 Hz, 4.00 Hz, pyrrole C_4_-H), 7.15 (1H, d, *J* = 3.60 Hz, furan C_4_-H), 7.22 (1H, d, *J* = 3.60 Hz, furan C_3_-H), 7.41 (1H, bs, pyrrole C_3_-H), 7.43–7.45 (2H, m, pyrrole C_5_-H, -CO-C*H*=CH-), 7.55-7.61 (3H, m, -CO-CH=C*H*-, phenyl C_3_-H, C_5_-H), 8.14 (2H, d, *J* = 8.80 Hz phenyl C_2_-H, C_6_-H). ^13^C-NMR (100 MHz, *δ* ppm, DMSO-*d*_6_): 37.17 (*C*H_3_), 108.23 (*C*H), 112.88 (*C*H), 117.87 (*C*H), 120.18 (*C*H), 122.83 (*C*H), 124.31 (*2C*H), 124.85 (*2C*H), 125.88 (*C*H), 131.38 (*C*H), 132.82 (*C*), 135.13 (*C*), 146.36 (*C*), 152.49 (2*C*), 177.81 (*C*). HRMS (ESI) (*m*/*z*): [M + H]^+^ calcd. for C_18_H_14_N_2_O_4_: 323.1026, found: 323.1017.

*1-(1-Methyl-1H-pyrrol-2-yl)-3-(5-(3-nitrophenyl)furan-2-yl)prop-2-en-1-one* (**2**). Yield: 57%; m.p. 123–124 °C. IR ν_max_ (cm^−1^): 3105.39 (aromatic C–H stretching), 2938.32 (aliphatic C–H stretching), 1645.28 (C=O stretching), 1589.34, 1506.41, 1458.18 (C=C stretching), 1402.25, 1346.31, 1247.94, 1215.15, 1093.64, 1064.71, 1039.63 (C–N and C–O stretching), 991.41, 968.27, 898.83, 864.11, 798.53, 785.03, 740.67, 682.80 (aromatic C–H out-of-plane bending). ^1^H-NMR (400 MHz, *δ* ppm, DMSO-*d*_6_): 3.97 (3H, s, CH_3_), 6.24 (1H, s, pyrrole C_4_-H), 7.15 (1H, d, *J* = 3.20 Hz, furan C_4_-H), 7.24 (1H, d, *J* = 3.20 Hz furan C_3_-H), 7.44 (1H, d, *J* = 3.20 Hz, pyrrole C_3_-H), 7.43–7.47 (2H, m, pyrrole C_5_-H, -CO-C*H*=CH-), 7.58 (1H, d, *J* = 15.60 Hz, -CO-CH=C*H*-), 7.74–7.82 (2H, m, phenyl C-H), 8.16–8.19 (2H, m, phenyl C-H). ^13^C-NMR (100 MHz, *δ* ppm, DMSO-*d*_6_): 37.16 (*C*H_3_), 108.20 (*C*H), 110.77 (*C*H), 117.65 (*C*H), 119.14 (*C*H), 120.08 (*C*H), 122.22 (*C*H), 123.83 (*C*H), 126.70 (*C*H), 130.10 (*C*H), 130.55 (*C*H), 130.84 (*C*H), 130.96 (*C*), 131.38 (*C*), 132.69 (*C*), 147.96 (*C*), 152.79 (*C*), 178.01 (*C*). HRMS (ESI) (*m*/*z*): [M + H]^+^ calcd. for C_18_H_14_N_2_O_4_: 323.1026, found: 323.1018.

*1-(1-Methyl-1H-pyrrol-2-yl)-3-(5-(2-nitrophenyl)furan-2-yl)prop-2-en-1-one* (**3**). Yield: 76%; m.p. 94–95 °C. IR ν_max_ (cm^−1^): 3097.68 (aromatic C-H stretching), 2939.42 (aliphatic C-H stretching), 1635.64 (C=O stretching), 1577.77, 1525.69, 1454.33 (C=C stretching), 1404.18, 1379.10, 1282.66, 1238.30, 1217.08, 1095.57, 1068.56, 1041.56 (C–N and C–O stretching), 981.77, 958.62, 923.90, 846.75, 815.89, 777.31, 744.52, 723.31, 682.80 (aromatic C–H out-of-plane bending). ^1^H-NMR (400 MHz, *δ* ppm, DMSO-*d*_6_): 3.93 (3H, s, CH_3_), 6.21 (1H, s, pyrrole C_4_-H), 7.10 (1H, d, *J* = 4.80 Hz, furan, C_4_-H), 7.11 (1H, d, *J* = 4.80 Hz, furan, C_3_-H), 7.19–7.21 (2H, m, pyrrole C_3_-H, C_5_-H), 7.32 (1H, d, *J* = 15.20 Hz, -CO-C*H*=CH-), 7.38 (1H, d, *J* = 15.60 Hz, -CO-CH=C*H*-), 7.59 (1H, t, *J* = 7.60 Hz, 15.20 Hz, phenyl C_4_-H), 7.73 (1H, t, *J* = 7.60 Hz, 15.20 Hz, phenyl C_5_-H), 7.89 (1H, d, *J* = 7.60 Hz, phenyl C_3_-H), 7.99 (1H, d, *J* = 8.00 Hz, phenyl C_6_-H). ^13^C-NMR (100 MHz, *δ* ppm, DMSO-*d*_6_): 37.14 (*C*H_3_), 108.31 (*C*H), 112.46 (*C*H), 116.89 (*C*H), 119.37 (*C*H), 121.55 (*C*H), 122.27 (*C*H), 123.70 (*C*H), 126.43 (CH), 128.50 (*C*H), 129.63 (*C*H), 131.36 (*C*H), 132.15 (*C*), 132.74 (*C*), 146.99 (*C*), 149.27 (*C*), 152.29 (*C*), 177.63 (*C*). HRMS (ESI) (*m*/*z*): [M + H]^+^ calcd. for C_18_H_14_N_2_O_4_: 323.1026, found: 323.1018.

*1-(1-Methyl-1H-pyrrol-2-yl)-3-(5-(2-nitro-4-chlorophenyl)furan-2-yl)prop-2-en-1-one* (**4**). Yield: 64%; m.p. 133–134 °C. IR ν_max_ (cm^−1^): 3109.25, 3066.82 (aromatic C-H stretching), 2939.12 (aliphatic C–H stretching), 1643.35 (C=O stretching), 1587.42, 1525.69, 1456.26 (C=C stretching), 1402.25, 1379.10, 1298.09, 1278.81, 1240.23, 1215.15, 1093.64, 1064.71, 1035.77, 1022.27 (C–N and C–O stretching), 987.55, 962.48, 925.83, 875.68, 819.75, 798.53, 738.74, 707.88, 686.66 (aromatic C–H out-of-plane bending). ^1^H-NMR (400 MHz, *δ* ppm, DMSO-*d*_6_): 3.97 (3H, s, CH_3_), 6.26 (1H, dd, *J* = 2.80 Hz, 4.40 Hz, pyrrole C_4_-H), 7.11 (1H, d, *J* = 3.60 Hz, furan C_4_-H), 7.17 (1H, d, *J* = 3.60 Hz, furan C_3_-H), 7.22–7.25 (2H, m, pyrrole C_3_-H, C_5_-H), 7.36 (1H, d, *J* = 15.60 Hz, -CO-C*H*=CH-), 7.41 (1H, d, *J* = 15.20 Hz, -CO-CH=C*H*-), 7.82 (1H, dd, *J* = 2.00 Hz, 8.40 Hz phenyl C_5_-H), 8.05 (1H, d, *J* = 8.80 Hz, phenyl C_6_-H), 8.16 (1H, d, *J* = 2.00 Hz, phenyl C_3_-H). ^13^C-NMR (100 MHz, *δ* ppm, DMSO-*d*_6_): 37.14 (*C*H_3_), 108.32 (*C*H), 113.04 (*C*H), 116.89 (*C*H), 119.37 (*C*H), 120.14 (*C*H), 122.56 (*C*H), 123.62 (*C*H), 126.23 (CH), 129.68 (*C*H), 131.34 (*C*H), 132.00 (*C*), 132.80 (*C*), 133.42 (C), 146.97 (*C*), 148.14 (*C*), 152.52 (*C*), 177.51 (*C*). HRMS (ESI) (*m*/*z*): [M + H]^+^ calcd. for C_18_H_13_ClN_2_O_4_: 357.0637, found: 357.0624.

*1-(1-Methyl-1H-pyrrol-2-yl)-3-(5-(2-chlorophenyl)furan-2-yl)prop-2-en-1-one* (**5**). Yield: 71%; m.p. 95–96 °C. IR ν_max_ (cm^−1^): 3128.54, 3068.75 (aromatic C–H stretching), 2939.52 (aliphatic C-H stretching), 1641.42 (C=O stretching), 1581.63, 1523.76, 1465.90 (C=C stretching), 1404.16, 1377.17, 1334.74, 1290.38, 1253.73, 1236.37, 1215.15, 1093.64, 1068.56, 1028.06 (C–N and C–O stretching), 981.77, 958.62, 921.97, 842.89, 761.88, 719.45, 682.80, 646.15 (aromatic C–H out-of-plane bending). ^1^H-NMR (400 MHz, *δ* ppm, DMSO-*d*_6_): 3.98 (3H, s, CH_3_), 6.23 (1H, s, pyrrole C_4_-H), 7.16 (1H, d, *J* = 3.60 Hz, furan C_4_-H), 7.24 (1H, bs, furan C_3_-H), 7.32 (1H, d, *J* = 4.00 Hz, pyrrole C_3_-H), 7.39–7.43 (2H, m, pyrrole C_5_-H, -CO-C*H*=CH-), 7.47–7.55 (2H, m, -CO-CH=C*H*-, phenyl C_4_-H), 7.60 (2H, d, *J* = 8.80 Hz, phenyl C_3_-H, C_5_-H), 8.16 (1H, dd, *J* = 1.60 Hz, 8.00 Hz, phenyl C_6_-H). ^13^C-NMR (100 MHz, *δ* ppm, DMSO-*d*_6_): 37.15 (*C*H_3_), 108.18 (*C*H), 113.74 (*C*H), 117.29 (*C*H), 119.95 (*C*H), 122.11 (*C*H), 126.77 (*C*H), 127.58 (*C*H), 127.63 (CH), 128.50 (*C*H), 129.39 (*C*H), 129.54 (*C*H), 130.82 (*C*), 131.38 (*C*), 132.64 (*C*), 150.90 (*C*), 151.04 (*C*), 177.96 (*C*). HRMS (ESI) (*m*/*z*): [M + H]^+^ calcd. for C_18_H_14_ClNO_2_: 312.0786, found: 312.0777. 

*1-(1-Methyl-1H-pyrrol-2-yl)-3-(5-(3-chlorophenyl)furan-2-yl)prop-2-en-1-one* (**6**). Yield: 82%; m.p. 121–122 °C. IR ν_max_ (cm^−1^): 3120.82, 3043.67 (aromatic C–H stretching), 2931.80, 2833.43 (aliphatic C-H stretching), 1643.35 (C=O stretching), 1587.42, 1550.77, 1527.62, 1460.11 (C=C stretching), 1404.18, 1379.10, 1328.95, 1294.24, 1240.23, 1211.30, 1099.43, 1068.56, 1026.13 (C–N and C–O stretching), 987.53, 964.41, 933.55, 887.26, 850.61, 785.03, 742.59, 725.23, 684.73 (aromatic C–H out-of-plane bending). ^1^H-NMR (400 MHz, *δ* ppm, DMSO-*d*_6_): 3.97 (3H, s, CH_3_), 6.23 (1H, s, pyrrole C_4_-H), 7.12 (1H, d, *J* = 3.60 Hz, furan C_4_-H), 7.23 (1H, bs, furan C_3_-H), 7.29 (1H, d, *J* = 4.00 Hz, pyrrole C_3_-H), 7.41–7.44 (1H, m, pyrrole C_5_-H), 7.48–7.67 (4H, m, -CO-C*H*=C*H*-, phenyl C-H), 7.83–7.94 (1H, m, phenyl C-H), 8.01 (1H, bs, phenyl C_2_-H). ^13^C-NMR (100 MHz, *δ* ppm, DMSO-*d*_6_): 37.18 (*C*H_3_), 108.16 (*C*H), 110.21 (*C*H), 117.82 (*C*H), 120.08 (*C*H), 121.74 (*C*H), 122.73 (*C*H), 123.64 (*C*H), 126.61 (*C*H), 128.02 (*C*H), 129.31 (*C*H), 130.77 (*C*H), 131.38 (*C*), 132.61 (*C*), 133.91 (*C*), 151.53 (*C*), 153.25 (*C*), 178.04 (*C*). HRMS (ESI) (*m*/*z*): [M + H]^+^ calcd. for C_18_H_14_ClNO_2_: 312.0786, found: 312.0772. 

*1-(1-Methyl-1H-pyrrol-2-yl)-3-(5-(4-chlorophenyl)furan-2-yl)prop-2-en-1-one* (**7**). Yield: 75%; m.p. 130–131 °C. IR ν_max_ (cm^−1^): 3113.11, 3043.67 (aromatic C–H stretching), 2939.34 (aliphatic C–H stretching), 1643.35 (C=O stretching), 1587.42, 1552.70, 1525.69, 1475.54 (C=C stretching), 1404.18, 1379.10, 1294.24, 1244.09, 1211.30, 1093.64, 1062.78, 1041.56, 1028.06 (C–N and C–O stretching), 985.62, 964.41, 921.97, 850.61, 790.81, 785.63, 744.52, 684.73 (aromatic C–H out-of-plane bending). ^1^H-NMR (400 MHz, *δ* ppm, DMSO-*d*_6_): 3.97 (3H, s, CH_3_), 6.23 (1H, dd, *J* = 2.40 Hz, 4.00 Hz, pyrrole C_4_-H), 7.11 (1H, d, *J* = 3.20 Hz, furan C_4_-H), 7.21 (1H, d, *J* = 3.60 Hz, furan C_3_-H), 7.23 (1H, bs, pyrrole C_3_-H), 7.43–7.47 (2H, m, pyrrole C_5_-H, -CO-C*H*=CH-), 7.53–7.59 (3H, m, -CO-CH=C*H*-, phenyl C_3_-H, C_5_-H), 7.95 (2H, d, *J* = 8.80 Hz, phenyl C_2_-H, C_6_-H). ^13^C-NMR (100 MHz, *δ* ppm, DMSO-*d*_6_): 37.18 (*C*H_3_), 108.16 (*C*H), 109.55 (*C*H), 117.91 (*C*H), 119.91 (*C*H), 121.43 (*C*H), 125.89 (*2C*H), 126.68 (*C*H), 128.29 (*C*H), 128.97 (*2C*H), 131.43 (*C*), 132.58 (*C*), 132.81 (*C*), 151.27 (*C*), 153.73 (*C*), 177.89 (*C*). HRMS (ESI) (*m*/*z*): [M+H]^+^ calcd. for C_18_H_14_ClNO_2_: 312.0786, found: 312.0772.

*1-(1-Methyl-1H-pyrrol-2-yl)-3-(5-(2,4-dichlorophenyl)furan-2-yl)prop-2-en-1-one* (**8**). Yield: 79%; m.p. 98–99 °C. IR ν_max_ (cm^−1^): 3107.32 (aromatic C–H stretching), 2943.37, 2843.07 (aliphatic C–H stretching), 1649.14 (C=O stretching), 1597.06, 1548.84, 1529.55, 1510.26, 1460.11 (C=C stretching), 1408.04, 1394.53, 1236.37, 1116.78, 1068.56, 1028.06 (C–N and C–O stretching), 991.41, 974.05, 921.97, 862.18, 798.53, 723.31, 688.59 (aromatic C–H out-of-plane bending). ^1^H-NMR (400 MHz, *δ* ppm, DMSO-*d*_6_): 3.96 (3H, s, CH_3_), 6.23 (1H, dd, *J* = 2.80 Hz, 4.40 Hz, pyrrole C_4_-H), 7.16 (1H, d, *J* = 3.60 Hz, furan C_4_-H), 7.24 (1H, d, *J* = 3.20 Hz, furan C_3_-H), 7.35 (1H, d, *J* = 3.60 Hz, pyrrole C_3_-H), 7.39 (1H, d, *J* = 4.00 Hz, pyrrole C_5_-H), 7.46 (1H, d, *J* = 15.60 Hz, -CO-C*H*=CH-), 7.56 (1H, d, *J* = 15.20 Hz, -CO-CH=C*H*-), 7.68 (1H, d, *J* = 3.60 Hz phenyl C-H), 7.93 (1H, d, *J* = 8.80 Hz, phenyl C-H), 8.17 (1H, d, *J* = 8.80 Hz, phenyl C-H). ^13^C-NMR (100 MHz, *δ* ppm, DMSO-*d*_6_): 37.16 (*C*H_3_), 108.20 (*C*H), 113.63 (*C*H), 114.16 (*C*H), 117.29 (*C*H), 120.03 (*C*H), 122.46 (*C*H), 126.61 (*C*H), 127.82 (CH), 128.11 (*C*H), 129.57 (*C*H), 130.19 (*C*), 130.40 (*C*), 131.35 (C), 149.89 (*C*), 151.30 (*C*), 153.18 (*C*), 177.89 (*C*). HRMS (ESI) (*m*/*z*): [M + H]^+^ calcd. for C_18_H_13_Cl_2_NO_2_: 346.0396, found: 346.0382. 

*1-(1-Methyl-1H-pyrrol-2-yl)-3-(5-(2,5-dichlorophenyl)furan-2-yl)prop-2-en-1-one* (**9**). Yield: 82%; m.p. 100–101 °C. IR ν_max_ (cm^−1^): 3070.68 (aromatic C-H stretching), 2943.37 (aliphatic C-H stretching), 1645.28 (C=O stretching), 1598.99, 1560.41, 1529.55, 1510.26, 1465.90 (C=C stretching), 1404.18, 1382.96, 1325.10, 1234.44, 1099.43, 1066.64, 1026.13 (C–N and C–O stretching), 995.27, 970.19, 885.33, 806.25, 783.10, 740.67, 690.52 (aromatic C–H out-of-plane bending). ^1^H-NMR (400 MHz, *δ* ppm, DMSO-*d*_6_): 3.92 (3H, s, CH_3_), 6.18 (1H, bs, pyrrole C_4_-H), 7.12 (1H, d, *J* = 2.80 Hz, furan C_4_-H), 7.19 (1H, bs, furan C_3_-H), 7.34 (1H, d, *J* = 3.60 Hz, pyrrole C_3_-H), 7.37–7.46 (2H, m, pyrrole C_5_-H, -CO-C*H*=CH-), 7.54–7.56 (1H, m, -CO-CH=C*H*-), 7.58–7.63 (2H, m, phenyl C-H), 8.09 (1H, s, phenyl C-H). ^13^C-NMR (100 MHz, *δ* ppm, DMSO-*d*_6_): 37.17 (*C*H_3_), 108.22 (*C*H), 114.74 (*C*H), 117.16 (*C*H), 120.23 (*C*H), 122.86 (*C*H), 126.61 (*C*H), 127.39 (*C*H), 129.04 (*C*H), 131.37 (*C*H), 132.50 (*C*H), 132.74 (*C*), 149.49 (*C*), 151.68 (*2C*), 152.63 (*C*), 154.72 (*C*), 177.96 (*C*). HRMS (ESI) (*m*/*z*): [M + H]^+^ calcd. for C_18_H_13_Cl_2_NO_2_: 346.0396, found: 346.0380.

*1-(1-Methyl-1H-pyrrol-2-yl)-3-(5-(3,4-dichlorophenyl)furan-2-yl)prop-2-en-1-one* (**10**). Yield: 83%; m.p. 132–133 °C. IR ν_max_ (cm^−1^): 3116.97 (aromatic C–H stretching), 2937.59, 2835.36 (aliphatic C-H stretching), 1645.28 (C=O stretching), 1587.42, 1566.20, 1531.48, 1462.04 (C=C stretching), 1409.96, 1332.81, 1294.24, 1255.66, 1236.37, 1134.14, 1066.64, 1026.06 (C–N and C–O stretching), 989.48, 960.55, 935.48, 871.82, 792.74, 732.95, 719.45, 690.52, 673.16 (aromatic C–H out-of-plane bending). ^1^H-NMR (400 MHz, *δ* ppm, DMSO-*d*_6_): 3.94 (3H, s, CH_3_), 6.20 (1H, dd, *J* = 2.40 Hz, 4.00 Hz, pyrrole C_4_-H), 7.07 (1H, d, *J* = 4.00 Hz, furan C_4_-H), 7.19 (1H, bs, furan C_3_-H), 7.27 (1H, d, *J* = 3.60 Hz, pyrrole C_3_-H), 7.39 (1H, d, *J* = 4.00 Hz, pyrrole C_5_-H), 7.43–7.44 (1H, m, -CO-C*H*=CH-), 7.54 (1H, d, *J* = 15.20 Hz, -CO-CH=C*H*-), 7.66 (1H, dt, *J* = 3.60, 8.00, 8.40 Hz, phenyl C-H), 7.86 (1H, dd, *J* = 2.00, 8.00 Hz, phenyl C-H), 8.13 (1H, d, *J* = 2.00 Hz, phenyl C-H). ^13^C-NMR (100 MHz, *δ* ppm, DMSO-*d*_6_): 37.17 (*C*H_3_), 108.17 (*C*H), 110.71 (*C*H), 117.79 (*C*H), 120.12 (*C*H), 121.99 (*C*H), 124.13 (*C*H), 125.61 (*C*H), 126.69 (*C*H), 129.91 (*C*H), 131.06 (*C*H), 131.42 (*C*), 131.97 (*C*), 132.64 (*C*), 151.78 (*2C*), 152.32 (*C*), 178.00 (*C*). HRMS (ESI) (*m*/*z*): [M + H]^+^ calcd. for C_18_H_13_Cl_2_NO_2_: 346.0396, found: 346.0379.

### 3.2. Microbiology

#### 3.2.1. Strains and Growth Conditions

Compounds **1**–**10** were screened for their antimicrobial activity against gram-negative bacteria, gram-positive bacteria, and yeasts, listed in Table 1. Bacteria were grown in Mueller-Hinton Broth and yeasts were grown in Sabouraud Dextrose Broth (SDB) during experiments. Overnight cultures were prepared routinely by subculturing of stock culture onto Mueller-Hinton Agar for bacteria and Sabouraud Dextrose Agar for yeasts [28].

#### 3.2.2. Microdilution Assay to Determine MICs

Microdilution susceptibility assay was used for the determination of antibacterial and anticandidal efficacy of compounds **1**–**10**. Chloramphenicol and ketoconazole were used as positive controls for bacteria and yeasts, respectively. All experiments were designed according to the Clinical Laboratory Standards Institute (CLSI) [29,30]. All chemicals were dissolved in dimethyl sulfoxide (DMSO) to obtain stock solution (10 mg/mL). Two-fold serial dilutions (800, 400, 200...0.78 µg/mL) of the compounds were prepared from stock solutions with dH_2_O. 100 µL of each solution was transferred to 96-well microtiter plates and 100 µL of 10^6^ CFU/mL of bacteria and 2 × 10^3^ of CFU/mL yeast were added to related wells. As negative controls, wells including microorganisms without agents and wells including agents without microorganisms were used. The plates were kept at 35 °C for 18–20 h and the growth was determined with resazurin (20 mg/mL). Assays were carried out in triplicate [28].

#### 3.2.3. ATP Luminescence Assay

A model from BioTek Instruments Inc. combined with a BacTiter-Glo^TM^ Microbial Cell Viability assay (Promega Corp., Madison, WI, USA) was used to provide a sensitive and rapid method for determining the number of viable microbial cells in culture based on quantitation of the ATP present. Cultures were treated with reference drugs at MIC values for 24 h. After incubation, cultures were added into the wells (final concentration of bacteria was 5 × 10^5^ CFU/mL and 2.5 × 10^6^ CFU/mL for yeasts). After adding the BacTiter reagent, incubation time for bacteria and yeasts was 5 min and 15 min, respectively.

The assay generates a “glow-type” luminescent signal, produced by the luciferase reaction. Briefly, after chemicals and strains were incubated for 24 h, 100 µL of the culture was taken from each well and mixed with the same volume of the BacTiter-Glo™ reagent in a white, opaque-walled microtiter plate. Control wells containing medium without cells were prepared to obtain a value for background luminescence. Cells without compound were used as an ATP-positive control. The microplates were further incubated under agitation for 7 min. Bioluminescence was recorded in a multi-detection microplate reader (BioTek Synergy 2 Multi-Mode Microplate Reader, BioTek Instruments, Winooski, VT, USA). In Figure 3, results were given as percentage of untreated control, which is set at 100%. Experiments were performed in triplicate and each sample was measured in four replicates [31].

#### 3.2.4. Flow Cytometry

In order to investigate the effects of compounds **7** and **9** on the integrity of *C. krusei* cells, flow cytometry was used with BD cell viability kits. Live cells are impermeable to dyes such as PI since they have intact membranes. TO is a permeant dye and enters live and dead cells to varying degrees. The fluorescent signal from TO in viable cells allows their enumeration, even when debris in the cell preparation contaminates a scatter gate around the cells. Thus, the combination of these two dyes provides a rapid and reliable method for discriminating live and dead eukaryotic cells, including yeasts. All steps were performed according to manufacturers’ recommendations. Briefly, *Candida* cells were cultured at 35 °C in SDB until late log phase. The cells were centrifuged and washed with phosphate-buffered saline (PBS) at pH = 7.0. Suspensions containing 2 × 10^3^ cells/mL were incubated with compounds **7** and **9**, and ketoconazole as a reference drug, for 4 h at 35 °C, with shaking (200 r.p.m.). Concentrations of each drug (around the corresponding MIC breakpoints) were 25 µg/mL. Suspensions of treated and untreated cells were incubated for 30 min in the presence of 2 µL of PI and 2 µL of TO, in the dark, at room temperature. The cell suspensions were analysed in a BD Accuri C6 flow cytometer (Becton-Dickinson, Mansfield, MA, USA). The cell scattergram (forward scatter: FS, side scatter: SS), the autofluorescence (without fluorocrome), and the intensity of fluorescence at FL1 (green fluorescence, 525 nm) and FL2 (red fluorescence, 620 nm) were recorded using a logarithmic scale [32,33].

#### 3.2.5. Statistical Analysis of the Quantitative Data

For each quantitative assay, the values obtained with the nine strains were tested using analysis of variance (ANOVA). A *p*-value below 0.05 indicates that at least one of the strains has a mean that differs from the others. For those assays that yielded a *p*-value below 0.05, the Tukey and Scheffe test was performed as a *post-hoc* test. The significance level alpha was set at 0.05. The software IBM SPSS Statistics^®^ version 21 (Armonk, NY, USA) was used for statistical analysis. All values were represented as mean ± standard deviation (SD).

### 3.3. Cytotoxicity

The anticancer effects of compounds **1**–**10** on A549 human lung adenocarcinoma (ATCC^®^ CCL-185™), HepG2 human hepatocellular carcinoma (ATCC^®^ HB-8065™), and C6 rat glioma (ATCC^®^ CCL107™) cell lines were assessed using 3-(4,5-Dimethylthiazol-2-yl)-2,5-diphenyltetrazolium bromide (MTT) assay, whereas the selectivity of compounds **1**–**10** was tested using NIH/3T3 mouse embryonic fibroblast cell line (ATCC^®^ CRL-1658™, London, UK). All cell lines were incubated according to the supplier’s recommendations. The cells were seeded as 1 × 10^4^ cells into each well of the 96-well plates. MTT assay was performed as previously described [34,35]. The compounds were tested between the concentrations equal to 1 mM and 0.000316 mM (1.0, 0.316, 0.10, 0.0316, 0.01, 0.00316, 0.001, 0.000316 mM). Stock solutions of compounds were prepared in dimethyl sulfoxide (DMSO) and further dilutions were made with fresh culture medium. The final DMSO concentration was under 1% [34] and cisplatin was used as a positive control. Inhibition % was calculated for each concentration according to the formula below, and IC_50_ values were determined by plotting a dose–response curve of inhibition % versus compound concentrations tested. All experiments were performed in quadruplicate.

Inhibition % = 100 − [(OD_test compound_ − OD_blank_/OD_solvent control_ − OD_blank_)] × 100 (1)

SI values were also calculated according to the formula [24,25] below:

SI = IC_50_ value for NIH/3T3 cell line/IC_50_ value for A549, HepG2, or C6 cell lines

IC_50_ values higher than any value (>value) were accepted as the value itself to calculate SI.

### 3.4. Genotoxicity

An Ames MPF 98/100 mutagenicity assay sample kit (Xenometrix AG, Allschwil, Switzerland) was used to evaluate the genotoxicity of compounds **7** and **9** as described previously [34,35]. *Salmonella typhimurium* TA98 (frameshift mutations) and TA100 (base-pair substitutions) strains were used and the compounds were tested in concentrations between 16 and 5000 μg/mL (5.0, 2.5, 1.25, 0.625, 0.3125, 0.156 mg/mL in DMSO) in accordance with the guidelines [36]. Mutagenity was tested with/without metabolic activation with an Aroclor™-1254 induced male Sprague–Dawley rat liver microsomal enzyme (S9) mix (Xenometrix AG, Allschwil, Switzerland). 2-Nitrofluorene (2.0 μg/mL) and 4-nitroquinoline *N*-oxide (0.1 μg/mL) were used without S9, and 1.0 μg/mL and 2.5 μg/mL of 2-aminoanthracene solutions were used with S9 against TA98 and TA100 as positive controls, respectively. At the end of each experiment, yellow wells were counted as positive and compared with the negative control. Fold induction over the negative control and fold induction over the baseline were calculated as described: Fold induction over the negative control is the ratio of the mean number of positive wells for the dose concentration divided by the mean number of positive wells for the zero-dose (negative) control. Fold induction over the baseline is the ratio of the mean number of positive wells for the dose concentration divided by the zero-dose baseline. The zero-dose baseline is obtained by adding one standard deviation to the mean number of positive wells of the zero-dose control. If the baseline is less than one, the value is set to one for calculation.

Genotoxicity was determined according to the previous criteria [37]. For a baseline value of ≤3, significant increases between 2- and 3-fold of the baseline were classified as weak mutagens, and increases ≥3-fold of the baseline were classified as mutagens. For a baseline value of >3, significant increases between 1.5- and 2.5-fold of the baseline were classified as weak mutagens, and increases ≥2.5-fold of the baseline were classified as mutagens. At least two adjacent doses with significant increases, or a significant increase at the highest dose level, should be observed for a mutagenic compound. All of the doses were compared according to Student’s *t*-test at *p* < 0.05 for statistical significance. Compounds which did not show any of the characteristics mentioned above were classified as non-mutagenic.

## 4. Conclusions

In this study, new pyrrole-based chalcone derivatives were synthesized and investigated for their antimicrobial effects on gram-negative bacteria, gram-positive bacteria, and *Candida* species, and their cytotoxic effects against A549 human lung adenocarcinoma, HepG2 human hepatocellular carcinoma, C6 rat glioma, and NIH/3T3 mouse embryonic fibroblast cell lines.

ATP luminescence assay indicated that compounds **7** and **9** showed significant antifungal activity against *C. krusei* in this series, therefore compounds **7** and **9** were chosen for flow cytometry analysis and Ames MPF assay. Flow cytometry analysis revealed that the percentage of dead cells treated with compound **7** was more than that treated with compound **9** and ketoconazole. According to Ames MPF assay, compounds **7** and **9** did not show any genotoxic activity. On the other hand, compounds **1** and **3** were found to be the most promising anticancer agents in this series against A549 and HepG2 cell lines, respectively. According to MTT assay, compounds **1** and **3** stand out as potential candidates for further studies.

## Figures and Tables

**Table 1:** Antimicrobial activity of compounds **1**–**10**.

Compound	MIC Values in μg/mL								
	Bacteria *					Yeasts **			
	1	2	3	4	5	6	7	8	9
**1**	200	200	100	100	200	100	200	100	200
**2**	200	100	100	100	200	100	100	100	100
**3**	100	200	100	100	200	50	100	50	100
**4**	100	200	200	100	100	200	100	50	200
**5**	400	200	200	400	200	200	200	100	200
**6**	200	200	200	100	200	200	200	100	200
**7**	400	200	100	400	100	50	100	100	25
**8**	400	400	200	100	200	200	100	100	50
**9**	200	400	200	100	200	200	100	50	25
**10**	200	100	400	100	200	100	200	100	200
**Control**	25	100	50	25	50	50	100	50	50

* **1**: *S. aureus* (ATCC^®^ 25923™), **2**: *E. faecalis* (ATCC^®^ 51299™), **3**: *E. faecalis* (ATCC^®^ 29212™), **4**: *E. coli* (ATCC^®^ 25922™), **5**: *K. pneumoniae* (ATCC^®^ 700603™); **Control**: Chloramphenicol for bacteria. ** **6**: *C. albicans* (ATCC^®^ 90028™), **7**: *C. glabrata* (ATCC^®^ 90030™), **8**: *C. parapsilosis* (ATCC^®^ 22019™), **9**: *C. krusei* (ATCC^®^ 6258™); **Control**: Ketoconazole for yeasts.

**Table 2:** The percentage of live and dead cells after incubation with the compounds.

	Live Cell %	Dead Cell %
Antibiotic-free control	98.9	0.1
Ketoconazole-treated cell	81.2	18.4
Compound **7**-treated cell	79.6	20.0
Compound **9**-treated cell	83.9	15.9

**Table 3:** IC_50_ values of compounds **1**–**10**.

Compound	IC_50_ (μg/mL)			
	HepG2 Cell Line	C6 Cell Line	A549 Cell Line	NIH/3T3 Cell Line
**1**	322	84	0.3	109
**2**	277	258	322	>322
**3**	27	>322	>322	>322
**4**	100	>357	>357	36
**5**	31	>311	311	45
**6**	98	103	98	38
**7**	23	311	98	40
**8**	106	90	225	277
**9**	101	109	>346	109
**10**	100	100	346	148
**Cisplatin**	38	46	19	>300

**Table 4:** SI values of compounds **1**–**10**.

Compound	SI Values		
	HepG2 Cell Line	C6 Cell Line	A549 Cell Line
**1**	0.339	1.298	363.33
**2**	1.162	1.248	1
**3**	11.926	1	1
**4**	0.360	0.100	0.100
**5**	1.450	0.145	0.145
**6**	0.388	0.369	0.388
**7**	1.739	0.129	0.408
**8**	2.613	3.077	1.231
**9**	1.079	1	0.315
**10**	1.480	1.480	0.428
**Cisplatin**	7.895	6.520	15.789

**Table 5:** Ames assay results of compounds **7** and **9**.

Compound	Concentrations (mg/mL)	REVERTANTS Fold Increase (Over Baseline)							
		Baseline		TA 98		Baseline		TA 100	
		S9+	S9−	S9+	S9−	S9+	S9−	S9+	S9−
**7**	0.16	3.95	3.04	0.68	0.66	5.91	3.00	0.56	0.44
	0.31			0.76	0.33			0.68	0.56
	0.63			1.01	0.44			0.79	0.67
	1.25			0.68	0.66			0.62	0.33
	2.5			0.51	0.33			0.90	0.33
	5			0.25	0.00 *			0.79	0.44
**9**	0.16	5.92	5.60	0.51	0.89	5.92	5.60	0.51	0.48
	0.31			0.79	0.83			0.73	0.54
	0.63			0.34 *	0.89			0.68	0.54
	1.25			0.39	1.07			0.68	0.54
	2.5			0.79	0.24 *			0.79	0.30 *
	5			0.51	0.54			0.90	0.12 *

* Significant increase *p* < 0.05 according to student’s *t*-test.
